# Myocardial fibrosis in primary aldosteronism

**DOI:** 10.3389/fendo.2025.1567876

**Published:** 2025-05-06

**Authors:** Yi-Lin Chen, Chi-Hua Chen, Xiao-Fei Ye, Jian-Zhong Xu, Li-Min Zhu, Ting-Yan Xu, Yan Li, Ji-Guang Wang

**Affiliations:** ^1^ Department of Cardiovascular Medicine, National Research Center for Translational Medicine, Shanghai Key Laboratory of Hypertension, The Shanghai Institute of Hypertension, Ruijin Hospital, Shanghai Jiaotong University School of Medicine, Shanghai, China; ^2^ Department of Radiology, Ruijin Hospital, Shanghai Jiaotong University School of Medicine, Shanghai, China; ^3^ School of Public Health, Shanghai Jiaotong University School of Medicine, Shanghai, China

**Keywords:** primary aldosteronism, myocardial fibrosis, late gadolinium enhancement, extracellular volume, metabolomic analysis

## Abstract

**Objective:**

We investigated myocardial fibrosis in relation to clinical and biochemical characteristics and the metabolomics in patients with primary aldosteronism.

**Methods:**

Our study included 54 patients with primary aldosteronism. We performed cardiac magnetic resonance to evaluate focal replacement myocardial fibrosis defined as late gadolinium enhancement (LGE) and diffuse interstitial fibrosis as assessed by extracellular volume (ECV) with T1 mapping. We collected information on demographics and data of clinical biomarkers, and performed echocardiography and metabolomic analysis.

**Results:**

Patients with LGE (n=30), compared with those without LGE (n=24), had a longer duration of hypertension, higher 24-hour, daytime and nighttime systolic blood pressure, left ventricular mass index, and plasma NT-proBNP (*P*<0.001). However, they had comparable T1 mapping measurements including ECV. LGE significantly (*P ≤* 0.007) and positively correlated with the duration of hypertension, ambulatory systolic blood pressure and LVMI, while ECV and native T1 were significantly (*P ≤* 0.027) and inversely associated with plasma renin activity and positively associated with aldosterone-to-renin ratio. Besides, both LGE and ECV significantly (*P ≤* 0.030) and positively correlated with NT-proBNP. Non-targeted metabolomic analysis showed that the amino-acid metabolism, especially the L-glutamate metabolism, significantly differed between patients with LGE and those without LGE and correlated with blood pressure and echocardiographic measurements.

**Conclusion:**

In patients with primary aldosteronism, focal replacement fibrosis was associated with cardiac afterload factors such as blood pressure, while diffuse interstitial fibrosis was associated with hyperaldosteronism. The amino-acid metabolism, especially the L-glutamate metabolism, might be involved in the process of myocardial fibrosis.

## Introduction

Primary aldosteronism is one of the most common secondary causes of hypertension, is characterized by renin-independent aldosterone production that may cause myocardial fibrosis and eventually cardiac structural and functional abnormalities (1–3). Since Brilla and Weber et al. first demonstrated myocardial fibrosis induced by hyperaldosteronism in the early 1990s (4, 5), several subsequent studies in patients with primary aldosteronism showed cardiac structural and functional dysfunctions (6–9) and other target organ damages such as microalbuminuria and aortic ectasia (10). Myocardial fibrosis can be detected non-invasively with cardiac magnetic resonance (MR) (11). Several previous cardiac MR studies have shown that myocardial fibrosis significantly increases the risk of cardiovascular events, independent of other cardiovascular risk factors (12).

According to the cardiomyopathic process, myocardial fibrosis can be classified as replacement, diffuse interstitial and infiltrative interstitial fibrosis. The first two types of fibrosis are usually presented in hypertensive patients and could be evaluated separately by late gadolinium enhancement (LGE) and extracellular volume fraction (ECV) with cardiac MR (12, 13). Recent studies have shown that the prevalence of replacement and diffuse interstitial myocardial fibrosis was higher in patients with primary aldosteronism than those with primary hypertension and normal controls (14, 15). However, it remains unclear on the risk factors of myocardial fibrosis in primary aldosteronism, especially with regard to the difference between the two types of fibrosis. Besides, the advancement of metabolomic analysis technology could detect small molecule compounds in metabolites and provide new perspectives for the investigation of disease pathogenesis (16). Therefore, we investigated myocardial fibrosis by the use of cardiac MR and its risk factors and potential metabolomic mechanisms in patients with primary aldosteronism.

## Methods

### Study population

We enrolled 56 consecutive patients with primary aldosteronism. All patients underwent a standardized diagnostic procedure. After mineralocorticoid receptor antagonists, angiotensin-converting enzyme inhibitors, angiotensin receptor blockers, β blockers, and diuretics were withdrawn for at least four weeks, plasma aldosterone concentration (PAC, in pg/ml) and plasma renin activity (PRA, in ng/ml/h) were measured after overnight fast and 30-min rest in the supine and standing positions. If a ratio of PAC to PRA (Aldosterone-to-Renin Ratio, ARR) was higher than 240 pg/ml per ng/ml/h, a physiological saline infusion test was performed to confirm the diagnosis of primary aldosteronism. The cut-off value for the post-saline PAC was 100 pg/ml. An adrenal venous sampling procedure and adrenal computed tomography imaging were performed for the subtyping of primary aldosteronism and lateralization in whom adrenalectomy was considered with the selectivity and lateralization index of adrenal venous sampling without ACTH stimulation ≥ 2, respectively. All patients with aldosterone producing adenoma (APA) underwent unilateral adrenalectomy after lateralization and had their diagnosis confirmed histopathologically (17–19). Patients were excluded if they had other forms of secondary hypertension, ischemic heart disease, valvular heart disease, cardiomyopathy, pacemaker implantation, atrial fibrillation, or suboptimal echocardiographic windows. The study protocol was complied with the Declaration of Helsinki and approved by the Ethics Committee of Ruijin Hospital, Shanghai Jiao Tong University School of Medicine, Shanghai, China. All study participants provided informed written consent prior to enrollment.

### Conventional echocardiography and cardiac MR measurements

Patients underwent standard transthoracic echocardiography and cardiac MR measurements after primary aldosteronism was diagnosed.

During echocardiography, we performed 2D, M-mode, continuous-wave, pulsed-wave, and pulsed tissue Doppler measurements in the sinus rhythm using the Vivid E9 and E95 Echo-System (General Electric Medical Health, Milwaukee, WI, USA) equipped with a M5S transducer. Images of parasternal long axis view were recorded to measure left ventricular end-diastole diameter (LVEDD), interventricular septal (IVS), left ventricular posterior wall (LVPW) thickness to calculate left ventricular mass index (LVMI) as described previously (20). Left ventricular ejection fraction (LVEF) was calculated using the modified Biplane Simpson method. The left atrial size was represented by left atrial maximal volume measured at end-systole from 4- and 2-chamber views, and indexed by body mass index as left atrial volume index (LAVI). Pulsed-wave Doppler of mitral valve inflow in the four-chamber view was recorded for measuring the peak early (E) and atrial filling (A) velocity, and for calculating the ratio of E/A. E’ was measured as the average of the peak early diastolic velocity at the septal and lateral mitral annulus by pulsed tissue Doppler (21).

All electrocardiographic gating cardiac MR examinations were performed on a 3.0 Tesla MRI scanner (Ingenia, Philips Healthcare, The Netherlands). The default protocol included cine, LGE and native and contrast-enhanced T1 mapping. The cine and LGE sequences included 2-, 3-, 4-chamber and short-axis images covering the whole left ventricular myocardium, which were acquired according to the following standardized protocol: Cine: voxel: 2×2×8mm, field of view: 350×350×8mm, time repetition/time echo: 3.1/1.55ms, flip angle: 45°, matrix: 176×168, and sense factor: 2; LGE: voxel: 1.6×1.9×10mm, field of view: 300×300×10mm, time repetition/time echo: 6.1/3.0ms, flip angle: 25°, matrix: 188×135, and sense factor: 2. Native and enhanced T1 mapping were acquired by the Modified Look-Locker recovery sequence using ‘5s(3s)3s’ and ‘4s(1s)3s(1s)2s’ scheme, respectively (voxel: 2×2×8mm, field of view: 300×300×8mm, time repetition/time echo: 2.3/1.07ms, flip angle: 20°, and matrix: 152×150). Mapping image acquisition was in short-axis slices with the gap of 2mm covering the whole left ventricular myocardium. LGE and enhanced T1 mapping were performed 10-15 minutes after intravenous administration of gadolinium contrast agents (Magnevist, Bayer Healthcare Pharmaceuticals, Germany) of 0.2 mmol/kg.

**Figure 1:**
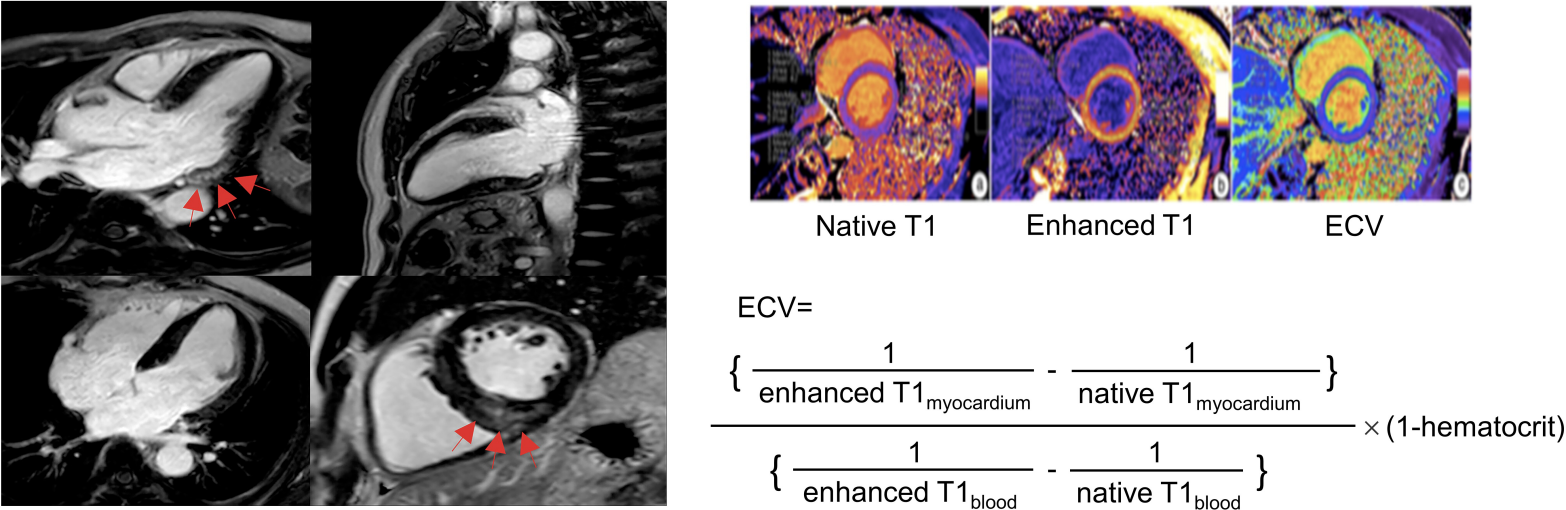
Late gadolinium enhancement (left) and extracellular volume fraction (right) analyses in primary aldosteronism. The cardiac fibrosis of late gadolinium enhancement on the left panel was indicated by the red arrow at basal inferolateral (upper left) and inferoseptal (bottom right) segments presented by focal high signal intensity in mid-layer myocardium on the left panel. Reproduced from ‘Chen YL, et al. Non-invasive left ventricular pressure-strain loop study on cardiac fibrosis in primary aldosteronism: A comparative study with cardiac magnetic resonance imaging. *Hypertension Research*. 2024. Springer Nature.’ (20).

Cardiac LGE imaging was analyzed on a commercial post-processing software QMass (Version 8.1, Medis Medical Imaging, The Netherlands). The evaluation of LGE was visually identified by two experienced radiologists and defined as areas with signal intensity > 5 SDs above remote normal myocardial region (**Figure 1**) (22). T1 mapping imaging including ECV values was acquired on a commercial post-processing software ECV (Version 8.1, Medis Medical Imaging, The Netherlands) by manually tracing endocardial and epicardial borders of left ventricular myocardium on short-axis mapping images while taking care to avoid imaging artifacts. The reference points were set at the superior and inferior left ventricular insertion to generate a 16-segment American Heart Association model. ECV values were generated by computing the pre- and post-contrast T1 values of the myocardium and blood pool together with hematocrit using the following formula: ECV = (1/enhanced T1_myocardium_ -1/native T1_myocardiaum_)/(1/enhanced T1_blood_ -1/native T1_blood_) × (1-hematocrit).

### Non-targeted metabolomic analysis

Serum samples in patients with (n=24) and without LGE (n=22) were analyzed using the ultra-high performance liquid chromatography-tandem mass spectrometry-based non-targeted metabolomics by Personalbio Technology Co., Ltd (Shanghai, China). Orthogonal partial least-square discriminant analysis (OPLS-DA) was used to evaluate the overall differences in the processed data between groups. A variable importance in projection (VIP) value > 1 and a *P* value < 0.05 (two-tailed Student’s t-test) were used to assess the significance of the differential expression of metabolites and screen the potentially relevant metabolic markers. Top 20 metabolites were selected using the Random Forest algorithm. The Kyoto Encyclopedia of Genes and Genomes (KEGG) was used to explore the related metabolic and signal transduction pathways of the significantly differentially expressed metabolites in primary aldosteronism patients. Benjamini-Hochberg false discovery rate (FDR) was further applied to the dataset with Q value < 0.05 considered significant.

### Statistical analysis

Statistical analyses were performed using the SPSS software version 22.0 (IBM Corp, Armonk, NY, USA). Continuous variables were expressed as mean ± SD or median (interquartile range) and analyzed using the one-way analysis of variance followed by LSD and Kruskal-Wallis test as appropriate. The Kolmogorov-Smirnov test was used to assess the distribution of continuous variables. Categorical variables were expressed as percentage and analyzed using the Chi square test. Multivariate logistic and linear regression analysis was performed to study the relationship of myocardial fibrosis. A *P* value less than 0.05 was considered statistically significant.

## Results

### Characteristics of the study patients

**Figure 2:**
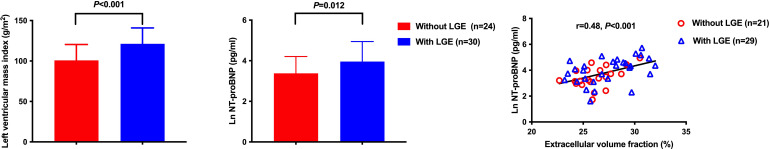
Myocardial fibrosis as an indicator of cardiac structure and function in primary aldosteronism. Both left ventricular mass index and NT-proBNP were significantly different in patients with and without late gadolinium enhancement. However, only NT-proBNP was significantly correlated with extracellular volume fraction.

Two of the 56 study patients were excluded from the present analysis, because of apical hypertrophic cardiomyopathy and suspected myocardial infarction. LGE was present in 30 of the 54 analyzed patients with a prevalence of 55.6%. **Table 1** shows the demographic and clinical characteristics in 30 patients with LGE and 24 patients without LGE. Though clinic blood pressure was similar between the two groups, 24-hour, daytime and nighttime systolic blood pressures were significantly higher in patients with LGE (*P ≤* 0.003). Patients with LGE had a significantly longer duration of hypertension and higher plasma NT-proBNP (*P ≤* 0.020, **Figure 2**). Other demographic and clinic characteristics were comparable between the two groups (*P*≥0.052). **Supplementary Table S1** shows the comparison between the two subtypes of primary aldosteronism, APA and idiopathic hyperaldosteronism (IHA). 5 patients could not be defined because of no or failed adrenal vein sampling. Almost all variables were comparable between the two subtypes except that PRA was significantly (*P ≤* 0.009) lower and ARR higher in APA than IHA.

**Table 1:** Demographic and clinical characteristics of patients with primary aldosteronism in the presence and absence of late gadolinium enhancement (LGE).

Characteristics	Without LGE (n=24)	With LGE (n=30)	*P* value
Age (years)	47.8 ± 8.4	52.0 ± 10.7	0.124
Male sex, (%)	15 (62.5)	22 (73.3)	0.623
Body mass index (kg/m^2^)	26.2 ± 2.6	26.7 ± 2.9	0.581
Clinic blood pressure (mmHg)			
Systolic	143.6 ± 12.1	147.1 ± 13.7	0.325
Diastolic	88.5 ± 9.0	86.0 ± 8.0	0.287
Ambulatory blood pressure (mmHg)			
24-hour systolic	132.1 ± 9.4	141.8 ± 11.1	0.001**
24-hour diastolic	85.3 ± 7.7	87.5 ± 8.9	0.347
Daytime systolic	134.8 ± 9.5	144.0 ± 11.9	0.003**
Daytime diastolic	87.1 ± 7.6	89.1 ± 9.0	0.406
Nighttime systolic	125.6 ± 10.8	136.4 ± 13.3	0.002**
Nighttime diastolic	80.4 ± 8.7	84.0 ± 9.9	0.177
Heart rate (beats/min)	71.1 ± 9.7	72.9 ± 9.8	0.491
Number of antihypertensive medications	2.5 (2.0, 3.0)	3.0 (2.8, 4.0)	0.052
Duration of hypertension (years)	6.0 (3.0, 10.0)	14.0 (10.0, 20.0)	0.001**
FBG (mmol/l)	5.5 ± 1.3	5.7 ± 1.1	0.449
TG (mmol/l)	1.6 ± 0.99	1.4 ± 0.65	0.741
TC (mmol/l)	4.7 ± 0.89	4.3 ± 0.76	0.155
NT-proBNP (pg/ml)	27.4 (19.6, 53.6)	63.0 (28.0, 107.5)	0.020*
PAC (pg/ml)	315 (221, 584)	306 (239, 547)	0.821
PRA (ng/ml/h)	0.38 (0.13, 0.98)	0.37 (0.17, 0.84)	0.938
ARR (pg/ml per ng/ml/h)	687 (399, 2035)	923 (307, 2555)	0.972
24-h urinary aldosterone excretion (μg)	28.2 (17.5, 37.2)	25.2 (16.6, 41.6)	0.986
Serum potassium concentration (mmol/l)	3.3 ± 0.4	3.3 ± 0.4	0.782
Serum creatinine concentration (μmol/l)	74.4 ± 15.9	80.1 ± 17.7	0.220

Values are mean ± SD, median (interquartile range) or percentage of patients (%). ARR, aldosterone-to-renin ratio. FBG, fasting blood glucose. NT-proBNP, N terminal pro B-type natriuretic peptide. PAC, plasma aldosterone concentration. PRA, plasma renin activity. TC, serum total cholesterol. TG, serum triglycerides.

*, *P*<0.05; **, *P*<0.01.

### Echocardiographic and T1 mapping measurements

**Table 2** shows the echocardiographic and T1 mapping measurements in the presence and absence of LGE. Patients with LGE had a significantly higher IVS, LVPW and LVMI than those without LGE (*P*<0.001, **Figure 2**). They had similar structural measurements such as LAVI, LVEDD, and LVESD, and similar diastolic functional parameters, such as E, E/A and E/e’ (*P*≥0.054). 50 patients were finally analyzed with the T1 mapping. The two groups were comparable in all measurements of T1 mapping including native T1, enhanced T1 and ECV (*P*≥0.115). **Supplementary Table S2** shows the comparison between the two subtypes of primary aldosteronism. Both subtypes were comparable in all variables including the prevalence of LGE and ECV (*P*≥0.097).

**Table 2:** Echocardiographic and T1 mapping measurements of patients with primary aldosteronism in the presence and absence of late gadolinium enhancement (LGE).

Variables	Without LGE	With LGE	*P* value
Echocardiographic measurements	n=24	n=30	
LAVI (ml/m^2^)	27.2 ± 7.0	29.9 ± 7.9	0.203
LVEDD (mm)	49.1 ± 4.7	51.6 ± 4.7	0.054
IVS (mm)	10.4 ± 1.2	11.8 ± 0.9	<0.001**
LVPW (mm)	10.1 ± 1.1	11.1 ± 0.8	<0.001**
LVMI (g/m^2^)	100.8 ± 19.6	121.3 ± 19.5	<0.001**
LVEF (%)	64.5 ± 4.1	66.3 ± 3.9	0.119
E (cm/s)	74.3 ± 16.7	73.9 ± 17.1	0.926
E/A	0.96 ± 0.26	0.90 ± 0.27	0.243
E/e’	9.6 ± 2.1	10.6 ± 2.9	0.334
T1 mapping measurements	n=21	n=29	
Native T1 (ms)	1333 ± 56	1321 ± 39	0.804
Enhanced T1 (ms)	548 ± 41	537 ± 37	0.463
ECV (%)	26.2 ± 1.9	27.4 ± 2.7	0.115

Values are mean ± SD. A, the peak atrial filling velocity of transmitral flow. E, the peak early filling velocity of tranmitral flow. e’, the average peak early filling velocity of septal and lateral mitral annulus. ECV, extracellular volume fraction. IVS, interventricular septum thickness. LAVI, left atrial volume index. LVEDD, left ventricular end-diastole diameter. LVEF, left ventricular ejection fraction. LVMI, left ventricular mass index. LVPW, left ventricular posterior wall thickness.

**, *P*<0.01.

### Correlation analysis of LGE and ECV

**Figure 3:**
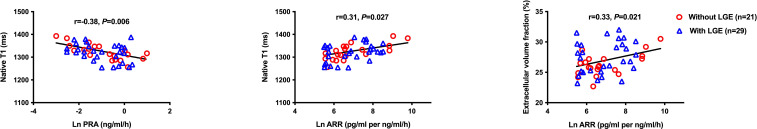
Correlation analysis of renin-angiotensin-aldosterone system indices with T1 mapping measurements. ARR, aldosterone-to-renin ratio; LGE, late gadolinium enhancement; PRA, plasma renin activity.

**Table 3** shows the results of the univariate logistic analysis on LGE in patients with primary aldosteronism. 24-hour, daytime and nighttime ambulatory, but not clinic, systolic blood pressure significantly correlated with the presence of LGE (*P*≤0.007). Other significant correlates of LGE included duration of hypertension, Ln NT-proBNP and LVMI (*P*≤0.03). In multivariate stepwise logistic regression analysis with age and sex forced in the model, nighttime systolic blood pressure, duration of hypertension and LVMI were still significantly associated with the presence of LGE (*P*≤0.03). Clinical biomarkers of the renin-angiotensin-aldosterone (RAAS) system were not correlated with replacement myocardial fibrosis (*P*≥0.90). On the other hand, unadjusted Pearson correlation analysis showed that Ln PRA and ARR significantly correlated with interstitial fibrosis indices such as native T1 and ECV (**Figure 3**). Besides, Ln NT-proBNP was also significantly correlated with ECV in both univariate (r=0.48, *P*<0.001, **Figures 2**) and multivariate analyses after adjusted for several clinical factors, such as sex, age, body mass index, and clinic blood pressure, with a standardized coefficient 0.53 (*P*<0.001).

**Table 3:** Univariate and multivariate logistic analyses on late gadolinium enhancement (LGE) in patients with primary aldosteronism (n=54).

Variables	Univariate		Multivariate	
	OR (95% CI)	*P* value	OR (95% CI)	*P* value
Age (years)	1.05 (0.99, 1.11)	0.124	0.97 (0.88, 1.08)	0.611
Sex, male	0.61 (0.19, 1.93)	0.396	2.22 (0.33, 14.90)	0.414
Body mass index (kg/m^2^)	1.06 (0.87, 1.29)	0.574		
Clinic blood pressure (mmHg)				
Systolic	1.02 (0.98, 1.07)	0.320		
Diastolic	0.97 (0.90, 1.03)	0.283		
Ambulatory blood pressure (mmHg)				
24-hour systolic	1.11 (1.03, 1.18)	0.004**		
24-hour diastolic	1.03 (0.97, 1.11)	0.343		
Daytime systolic	1.09 (1.02, 1.16)	0.007**		
Daytime diastolic	1.03 (0.96, 1.10)	0.400		
Nighttime systolic	1.09 (1.02, 1.16)	0.006**	1.14 (1.03, 1.27)	0.015*
Nighttime diastolic	1.05 (0.98, 1.12)	0.176		
Heart rate (beats/min)	1.02 (0.96, 1.08)	0.484		
Number of antihypertensive medications	1.82 (0.98, 3.37)	0.058		
Duration of hypertension (years)	1.17 (1.06, 1.30)	0.003**	1.20 (1.02, 1.41)	0.030*
Ln NT-proBNP (pg/ml)	2.03 (1.07, 3.86)	0.030*		
Ln PAC (pg/ml)	0.96 (0.40, 2.32)	0.927		
Ln PRA (ng/ml/h)	0.94 (0.60, 1.74)	0.940		
Ln ARR (pg/ml per ng/ml/h)	0.97 (0.61, 1.55)	0.896		
Ln 24-h urinary aldosterone excretion (μg)	0.97 (0.35, 2.65)	0.950		
LAVI (ml/m^2^)	1.05 (0.97, 1.14)	0.205		
LVMI (g/m^2^)	1.06 (1.02. 1.10)	0.003**	1.07 (1.02, 1.12)	0.007**
LVEF (%)	1.12 (0.97, 1.30)	0.124		
E/A	0.78 (0.52, 1.16)	0.222		
E/e’	1.17 (0.93, 1.48)	0.179		

ARR, aldosterone-to-renin ratio. A, the peak atrial filling velocity of transmitral flow. E, the peak early filling velocity of tranmitral flow. LAVI, left atrial volume index. LVEF, left ventricular ejection fraction. LVMI, left ventricular mass index. NT-proBNP, N terminal pro B-type natriuretic peptide. PAC, plasma aldosterone concentration. PRA, plasma renin activity.

*, *P*<0.05; **, *P*<0.01.

### Non-targeted metabolomic analysis

**Figure 4:**
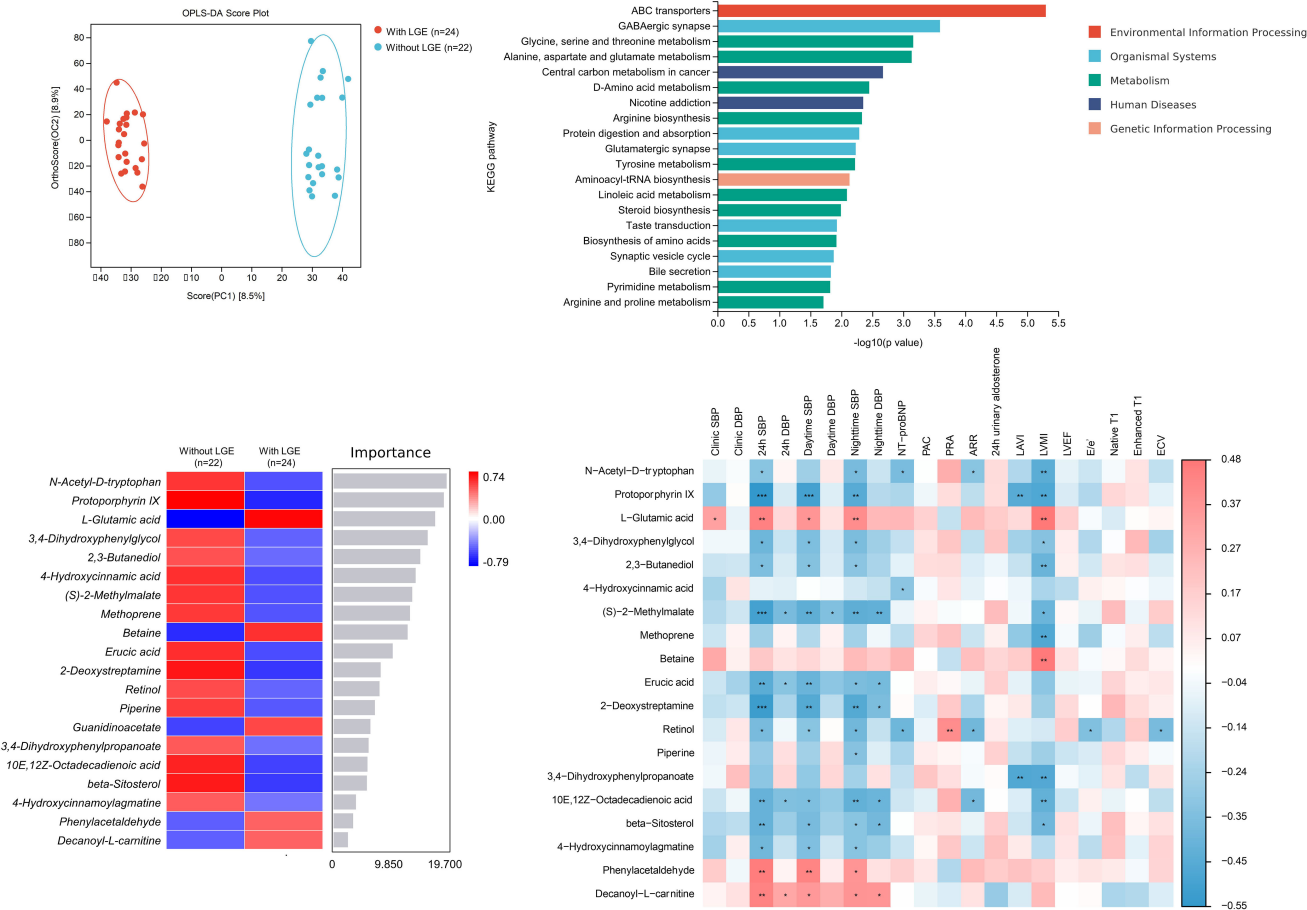
Non-targeted metabolomic analysis between patients with and without late gadolinium enhancement (LGE) in primary aldosteronism (OPLS-DA plot on the left upper panel, Top 20 KEGG pathway visualization on the right upper panel, Top 20 metabolites heatmap on the left lower panel and correlation heatmap between metabolites and clinical and imaging indices on the right lower panel). ARR, aldosterone-to-renin ratio; KEGG, Kyoto Encyclopedia of Genes and Genomes; LGE, late gadolinium enhancement; OPLS-DA, orthogonal partial least-square discriminant analysis; PAC, plasma aldosterone concentration. PRA, plasma renin activity. *, *P*<0.05. **, *P*<0.01. ***, *P*<0.001.

Totally, 49 metabolites with a VIP value > 1 and *P* value < 0.05 were selected significantly different in the presence and absence of LGE in patients with primary aldosteronism. **Figure 4**; **Supplementary Table S3** show the results of non-targeted metabolomic analysis on myocardial fibrosis in primary aldosteronism. OPLS-DA showed significant heterogeneity in the spatial distribution of samples between groups with and without LGE, suggesting the existence of significant metabolites between groups. Among the top 20 significant metabolites and KEGG pathways with visualization in **Figure 4**, the amino-acid metabolism significantly correlated with LGE, including the glutamate metabolism, threonine metabolism, D-amino acid metabolism, and so on. L-glutamate was one of the most significant metabolites and significantly correlated with LVMI and clinic, 24-hour, daytime and nighttime systolic blood pressure. However, few metabolites except retinol correlated with ECV.

## Discussion

Our study showed that different types of myocardial fibrosis had different correlates in patients with primary aldosteronism. Patients with focal replacement fibrosis had significantly higher ambulatory systolic blood pressure and longer duration of hypertension with more impaired left ventricular structure while diffuse interstitial fibrosis was more related to abnormalities in plasma renin and aldosterone. Moreover, the significant correlation between NT-proBNP and both LGE and ECV indicates that heart failure might involve both focal replacement and diffuse interstitial fibrosis in primary aldosteronism patients. The potential mechanism of myocardial fibrosis could possibly be related with amino-acid metabolism, especially that of L-glutamate.

Myocardial focal replacement fibrosis is usually considered to be the later stage of cardiomyocyte necrosis that may cause LGE with cardiac MR. Significantly longer duration of hypertension in patients with this type of myocardial fibrosis indicated the progressive disease burden on cardiomyocyte damage. The effect of long-term hypertension on the incidence of replacement fibrosis during follow-up was demonstrated previously in the Multi-Ethnic Study of Atherosclerosis (MESA) study (23). Besides, in another study by Vergaro G et al., duration of disease was also found significantly correlated with the presence of LGE (24). Though clinic blood pressure was similar between groups, ambulatory systolic blood pressure was significantly higher in patients with LGE than those without LGE. 24-hour ambulatory blood pressure is considered to be superior to clinic blood pressure in blood pressure monitoring (25). Progressive left ventricular remodeling arising from hypertension may lead to hypertrophied myocytes and increased left ventricular remodeling. The continuous myocardial overload coupled with impaired microvascular circulation might result in focal myocyte necrosis, leading to focal replacement fibrosis (26). Indeed, in our study, we found significant relationship of LGE with cardiac structure changes, such as LVMI, which could be explained by the pathophysiological process of myocardial fibrosis in hypertension.

NT-proBNP was significantly higher in patients with LGE and correlated with ECV. While focal replacement fibrosis could be detected by LGE, diffuse interstitial fibrosis, on the other hand, is seen in hypertensive patients with a diffuse distribution within the interstitium as well as the perivasculature (27). Being the important clinical biomarker of heart failure with preserved LVEF, the elevation of NT-proBNP was considered to reflect cardiac dysfunction. In previous studies, the abnormal metabolism of natriuretic peptide was also found significantly correlated with both of the two types of myocardial fibrosis (28, 29). In hypertensive patients, cardiac natriuretic peptides are secreted in response to elevated pressure load, followed by structural rearrangement of cardiac chambers and expansion of extracellular matrix, which in turn triggers the production of cardiac natriuretic peptides.

Significant correlation was found between T1 mapping measurements and ARR. Hypersecretion of aldosterone and inhibition of renin are the pathophysiology of primary aldosteronism, leading to abnormal elevation of ARR. The dysregulation of renin-angiotensin-aldosterone system (RAAS) could cause a maladaptive remodeling of the extracellular matrix, with disruption and loss of elastic fibers, altered collagen synthesis and fibrosis (30), thus leading to structural and functional alterations of the left heart in primary aldosteronism (31). Indeed, in the Atherosclerosis Risk in Communities study in 4547 participants without heart failure, Brown JM et al. also found that renin suppression and aldosterone excess were associated with cardiac structural and functional alterations independent of multiple clinical risk factors (32). The correlation between T1 mapping measurements and ARR in our study further confirmed the impact of RAAS disorder on the regulation of cardiac interstitial fibrosis. In a recent imaging study of cardiac MR, Redheuil A et al. found that only primary pathological aldosterone excess combined with high blood pressure increased both extracellular myocardial matrix and intracellular mass, while secondary aldosterone excess or primary hypertension did not affect extracellular myocardial matrix (33). Moreover, in a previous prospective clinical study, Rossi GP et al., found that such cardiac remodeling caused by hyperaldosteronism might be reversed by surgical or medical intervention targeting aldosterone excess (34).

Interestingly, though NT-proBNP were correlated with both replacement and interstitial myocardial fibrosis, other measurements were associated with only one or another myocardial fibrosis. We speculated that a possible explanation might be the different pathophysiology of these two types of fibrosis. Both types of fibrosis represent principal but distinct domains of cardiac vulnerability with diffuse interstitial fibrosis indicating architectural distortion mediated by extracellular matrix, and focal replacement fibrosis, on the other hand, indicating cardiomyocyte necrosis and damage (35). In other words, fibroblast plasticity might be the potential mechanism of diffuse interstitial fibrosis by producing extracellular matrix components and could be reversible when controlling the transition between fibroblast and myofibroblast (36). Focal replacement fibrosis, on the other hand, could be considered as the end stage of fibrosis and is irreversible. Measurements that were related to focal replacement fibrosis, such as LVMI and systolic blood pressure, reflect cardiac afterload and myocyte remodeling, while the abnormalities of RAAS leads to water and sodium retention and increase cardiac preload that affects diffuse interstitial fibrosis. The different impacts and pathophysiology between these two types of myocardial fibrosis might suggest different strategies, such as well blood pressure control in preventing focal replacement fibrosis and early interventions targeting aldosterone excess in diffuse interstitial fibrosis.

In metabolomic analysis, L-glutamate was one of the most significant metabolites between groups and correlated with several clinical and echocardiographic measurements. Previous studies have shown that glutaminolysis from glutamine to glutamate is involved in fibroblast proliferation and migration and associated with fibrosis in several organs, such as the heart, liver and lungs in animal models, and the progression might be alleviated by inhibiting its enzyme glutaminase (37–39). Lin ZR et al. found that glutamate dehydrogenase, the enzyme that catalyzed conversion of glutamate into α-ketoglutarate, participated in isoprenaline-induced cardiac hypertrophy through activating mammalian target of rapamycin signaling (40). Besides, there was also evidence that hypertension induced by angiotensin II was associated with glutamate signalling (41), and inhibition of this anaplerotic pathway could possibly contribute to the suppression of cardiac hypertrophy and fibrosis (42). Few metabolites except retinol were correlated with ECV among the top 20 metabolites. It was found in recent studies that this lipid-soluble vitamin was involved in ferroptosis, a programmed cell death caused by iron-dependent phospholipid peroxidation (43) and the deficiency of its downstream, all-trans retinoic acid, was mediated by retinol dehydrogenase 10 reduction and promoted diabetic cardiomyopathy in male mice (44). However, in the latter study, the disordered cardiac retinol metabolism was, on the contrary, characterized by retinol overload, which was opposite to the finding of our metabolomic analysis. Thus, the exact effect of retinol on myocardial fibrosis in primary aldosteronism patients might need to be further studied. The finding of these metabolites might provide novel indicators of myocardial fibrosis and new potential pathological mechanisms in primary aldosteronism.

Our study had several limitations. First, this was a cross-sectional study with a relatively small sample size. Thus, we could not draw any conclusion on the causal relationship of the myocardial fibrosis. Future clinical study with large sample size and with prospective design might be required to further establish its causal relationship, and investigate other possible fibrosis indicators, such as matrix metalloproteinase (MMP) and its inhibitor, tissue inhibitor of metalloproteinase (TIMP), in addition to NT-proBNP, so as to provide more information on the development of myocardial fibrosis. Second, due to the unsatisfied image quality, T1 mapping measurements were not possible to analyze in some study patients, which might further introduce bias. Third, the finding of metabolomic analysis could be regarded as an explorative study, which should further be confirmed with larger sample size and with animal experiments. Fourth, previous studies have shown significantly higher prevalence of LGE and higher ECV in patients with primary aldosteronism than normal controls and primary hypertensive patients (14, 15). Our study did not include these latter participants.

In conclusion, patients with primary aldosteronism had focal replacement and diffuse interstitial fibrosis, both of which correlated with the biological markers of heart failure. In addition, focal replacement fibrosis was associated with those that reflect cardiac afterload such as blood pressure and left ventricular mass index. Diffuse interstitial fibrosis might be affected by abnormalities in hyperaldosteronism. The amino-acid metabolism, especially the L-glutamate metabolism, might be involved in the process of myocardial fibrosis.

## Data Availability

The datasets presented in this article are not readily available because the data are not publicly available due to the containing information that could compromise the privacy of research participants. Requests to access the datasets should be directed to Ting-Yan Xu, xtyswallow@sina.com.
