# Supplementary material for: Myocardial fibrosis in primary aldosteronism
Source: Front Endocrinol (Lausanne). 2025 May 6;16:1567876. doi: 10.3389/fendo.2025.1567876 (PMC12088938; doi:10.3389/fendo.2025.1567876)
Supplement: Supplementary file 1 [file Table1.docx]

**Supplemental Material**

This Data Supplement has been provided by the authors to give readers additional information about their work.

Supplement to:

Chen YL, et al. Myocardial fibrosis in primary aldosteronism

**Table S1**. Demographic and clinical characteristics of primary aldosteronism patients with subtypes APA and IHA

**Table S2**. Echocardiographic and cardiac magnetic resonance measurements of primary aldosteronism patients with subtypes APA and IHA

**Table S3**. Significant metabolites of primary aldosteronism patients with and without late gadolinium enhancement (n=46)

**Table S1**. Demographic and clinical characteristics of primary aldosteronism patients with subtypes APA and IHA

| Characteristics | APA (n=31) | IHA (n=18) | *P* value |
| --- | --- | --- | --- |
| Age (years) | 49.9±9.5 | 49.9±11.4 | 0.942 |
| Male sex, (%) | 21 (67.7) | 13 (72.2) | 0.743 |
| Body mass index (kg/m^2^) | 26.4±2.8 | 26.6±2.9 | 0.796 |
| Clinic blood pressure (mmHg) | | | |
| Systolic | 145.5±12.1 | 145.6±13.6 | 0.966 |
| Diastolic | 89.1±7.9 | 84.8±8.6 | 0.084 |
| Ambulatory blood pressure (mmHg) | | | |
| 24-hour systolic | 137.9±10.7 | 136.6±13.7 | 0.709 |
| 24-hour diastolic | 87.5±9.1 | 84.8±7.2 | 0.286 |
| Daytime systolic | 140.1±10.3 | 140.4±15.2 | 0.938 |
| Daytime diastolic | 89.3±8.8 | 87.1±7.9 | 0.367 |
| Nighttime systolic | 133.1±13.7 | 127.0±11.7 | 0.119 |
| Nighttime diastolic | 83.7±10.3 | 79.1±6.9 | 0.101 |
| Heart rate (beats/min) | 73.0±10.9 | 70.1±8.5 | 0.325 |
| Number of antihypertensive medications | 3.0 (2.0, 3.0) | 3.0 (3.0, 4.0) | 0.104 |
| Duration of hypertension (years) | 10.0 (3.0, 15.0) | 10.0 (8.5, 17.0) | 0.533 |
| FBG (mmol/l) | 5.5±1.4 | 5.7±1.0 | 0.724 |
| TG (mmol/l) | 1.4±0.9 | 1.7±0.8 | 0.065 |
| TC (mmol/l) | 4.4±0.9 | 4.7±0.8 | 0.246 |
| NT-proBNP (pg/ml) | 40.3 (19.4, 84.5) | 36.9 (24.7, 57.2) | 0.926 |
| PAC (pg/ml) | 305.6 (216.7, 537.6) | 280.1 (237.7,562.8) | 0.694 |
| PRA (ng/ml/h) | 0.26 (0.12, 0.62) | 0.73 (0.37, 1.05) | 0.009** |
| ARR (pg/ml per ng/ml/h) | 1294 (495,2527) | 333 (286, 706) | 0.006** |
| 24-h urinary aldosterone excretion (μg) | 24.5 (17.6, 41.9) | 26.5 (12.0,31.5) | 0.378 |
| Serum potassium concentration (mmol/l) | 3.3±0.3 | 3.3±0.5 | 0.975 |
| Serum creatinine concentration (μmol/l) | 76.3±14.6 | 76.6±15.0 | 0.948 |

Values are mean ± SD, median (interquartile range) or percentage of patients (%). APA, aldosterone-producing adenoma. ARR, aldosterone-to-renin ratio. FBG, fasting blood glucose. IHA, idiopathic hyperaldosteronism. NT-proBNP, N terminal pro B-type natriuretic peptide. PAC, plasma aldosterone concentration. PRA, plasma renin activity. TC, total cholesterol. TG, total triglyceride.

**, *P*<0,01

**Table S2**. Echocardiographic and cardiac magnetic resonance measurements of patients with subtypes of primary aldosteronism

| Variables | APA | IHA | *P* value |
| --- | --- | --- | --- |
| Echocardiographic measurements | n=31 | n=18 |  |
| LAVI (ml/m^2^) | 28.2±7.8 | 28.8±5.4 | 0.413 |
| LVEDD (mm) | 50.6±4.6 | 50.7±5.6 | 0.977 |
| IVS (mm) | 11.2±1.3 | 11.1±1.3 | 0.614 |
| LVPW (mm) | 10.5±1.0 | 10.8±1.3 | 0.318 |
| LVMI (g/m^2^) | 112.3±22.8 | 112.1±24.0 | 0.977 |
| LVEF (%) | 65.5±4.1 | 65.7±3.6 | 0.472 |
| E (cm/s) | 71.2±15.6 | 79.6±18.2 | 0.097 |
| E/A | 0.89±0.23 | 1.02±0.32 | 0.206 |
| E/e' | 10.2±2.7 | 9.9±2.1 | 0.942 |
| CMR measurements | n=28# | n=17# |  |
| With LGE, (%) | 18 (58.1) | 9 (50.0) | 0.584 |
| Native T1 (ms) | 1330.1±40.0 | 1318.0±61.8 | 0.134 |
| Enhanced T1 (ms) | 537.7±36.7 | 541.5±44.7 | 0.754 |
| ECV (%) | 27.4±2.6 | 26.3±2.2 | 0.143 |

Values are mean ± SD. A, the peak atrial filling velocity of transmitral flow. APA, aldosterone-producing adenoma. E, the peak early filling velocity of tranmitral flow. e', the average peak early filling velocity of septal and lateral mitral annulus. ECV, extracellular volume fraction. IHA, idiopathic hyperaldosteronism. IVS, interventricular septum thickness. LAVI, left atrial volume index. LGE, late gadolinium enhancement. LVEDD, left ventricular end-diastole diameter. LVEF, left ventricular ejection fraction. LVMI, left ventricular mass index. LVPW, left ventricular posterior wall thickness.

#, sample size for the analysis of T1 mapping measurements including native T1, enhanced T1 and ECV.

**Table S3**. Significant metabolites of primary aldosteronism patients with and without late gadolinium enhancement (n=46)

|  | VIP | Log2 FC | *P* value | FDR | Class | KEGG pathway |
| --- | --- | --- | --- | --- | --- | --- |
| N-Acetyl-D-tryptophan | 1.79 | -3.67 | <0.001 | 0.01 | / | / |
| Protoporphyrin IX | 2.28 | -2.81 | <0.001 | <0.001 | Tetrapyrroles and derivatives | Metabolism of cofactors and vitamins |
| L-Glutamic acid | 3.21 | 1.39 | <0.001 | <0.001 | Carboxylic acids and derivatives | Amino acid metabolism |
| 3,4-Dihydroxyphenylglycol | 1.56 | -1.47 | 0.005 | 0.03 | Phenols | Amino acid metabolism |
| 2,3-Butanediol | 1.48 | -2.23 | 0.007 | 0.04 | Organooxygen compounds |  |
| 4-Hydroxycinnamic acid | 2.77 | -1.47 | <0.001 | 0.03 | Cinnamic acids and derivatives | Amino acid metabolism |
| (S)-2-Methylmalate | 2.02 | -1.22 | <0.001 | <0.001 | / | / |
| Methoprene | 2.00 | -1.38 | <0.001 | 0.001 | / | / |
| Betaine | 2.98 | 0.55 | <0.001 | 0.003 | Carboxylic acids and derivatives | Amino acid metabolism |
| Erucic acid | 2.09 | -1.33 | <0.001 | <0.001 | Fatty Acyls | Lipid metabolism |
| 2-Deoxystreptamine | 2.31 | -1.29 | <0.001 | <0.001 | / | Biosynthesis of other secondary metabolites |
| Retinol | 1.82 | -0.77 | <0.001 | 0.003 | / | Metabolism of cofactors and vitamins |
| Piperine | 1.81 | -2.36 | <0.001 | 0.007 | / | / |
| Guanidinoacetate | 2.65 | 0.49 | <0.001 | 0.01 | Carboxylic acids and derivatives | Amino acid metabolism |
| 3,4-Dihydroxyphenylpropanoate | 1.53 | -2.96 | 0.004 | 0.029 | Phenylpropanoic acids | Amino acid metabolism |
| 10E,12Z-Octadecadienoic acid | 2.24 | -1.41 | <0.001 | <0.001 | Fatty Acyls | Lipid metabolism |
| beta-Sitosterol | 2.31 | -0.94 | <0.001 | <0.001 | Steroids and steroid derivatives | Lipid metabolism |
| 4-Hydroxycinnamoylagmatine | 1.58 | -0.44 | 0.001 | 0.013 | Cinnamic acids and derivatives | Amino acid metabolism |
| Phenylacetaldehyde | 2.37 | 0.49 | 0.001 | 0.012 | Benzene and substituted derivatives | Amino acid metabolism |
| Decanoyl-L-carnitine | 1.72 | 2.92 | <0.001 | 0.005 | / | / |
| Cyclohexylamine | 1.72 | 0.47 | <0.001 | 0.006 | Organonitrogen compounds | / |
| Epsilon-caprolactam | 1.83 | 1.85 | <0.001 | 0.003 | Lactams | / |
| 3-Amino-4-hydroxybenzoate | 1.64 | 1.05 | <0.001 | 0.014 | / | / |
| Anabasine | 1.66 | -0.12 | <0.001 | 0.007 | / | / |
| Levonordefrin | 1.51 | 0.58 | 0.002 | 0.019 | Benzene and substituted derivatives |  |
| Vanylglycol | 1.78 | 1.29 | <0.001 | 0.006 | Phenols | Amino acid metabolism |
| (R)-4-Hydroxymandelate | 1.40 | -0.39 | 0.007 | 0.039 | / | / |
| (1S,2R,4S)-(-)-Bornyl acetate | 1.54 | -0.78 | 0.002 | 0.019 | / | / |
| Se-Methylselenocysteine | 1.65 | -0.51 | 0.001 | 0.011 | Carboxylic acids and derivatives | Metabolism of other amino acids |
| 3-Methyl-L-tyrosine | 1.61 | -0.21 | <0.001 | 0.008 | / | / |
| Dodecanoic acid | 1.88 | -0.07 | <0.001 | 0.003 | Fatty Acyls | Lipid metabolism |
| N5-(L-1-Carboxyethyl)-L-ornithine | 1.71 | -0.54 | <0.001 | 0.005 | / | / |
| Lumichrome | 1.53 | 0.15 | 0.005 | 0.033 | / | / |
| Pseudouridine | 1.81 | -0.20 | <0.001 | 0.004 | Nucleoside and nucleotide analogues | Nucleotide metabolism |
| OPEO | 1.63 | -0.97 | 0.001 | 0.012 | Extractables/Leachables; Textile Chemicals/Auxiliary/Dyes | / |
| (S)-Abscisic acid | 1.99 | -1.02 | 0.002 | 0.048 | Prenol lipids | / |
| Inosine | 2.05 | -1.02 | 0.002 | 0.045 | Purine nucleosides | Membrane transport; Nucleotide metabolism |
| Oleic acid | 1.96 | -1.59 | <0.001 | 0.002 | Fatty Acyls | Lipid metabolism |
| Indoleglycerol phosphate | 1.47 | -2.29 | 0.007 | 0.042 | / | Amino acid metabolism |
| 19(S)-HETE | 2.25 | 1.21 | <0.001 | 0.024 | Fatty Acyls | Lipid metabolism |
| Hesperetin | 1.77 | -0.99 | <0.001 | 0.003 | Flavonoids | / |
| 11-Dehydrocorticosterone | 2.14 | -1.33 | 0.001 | 0.033 | Steroids and steroid derivatives | Lipid metabolism |
| Rosmarinic acid | 1.64 | -1.19 | 0.002 | 0.014 | Cinnamic acids and derivatives | Amino acid metabolism |
| Misoprostol | 1.78 | -0.74 | <0.001 | 0.008 | / | / |
| Riboflavin | 1.51 | -0.13 | 0.003 | 0.020 | Pteridines and derivatives | Metabolism of cofactors and vitamins |
| Vitamin D3 | 1.56 | 0.31 | 0.005 | 0.034 | Steroids and steroid derivatives | Lipid metabolism |
| Allocholic acid | 1.46 | -0.39 | 0.005 | 0.036 | Steroids and steroid derivatives | Lipid metabolism |
| 3-Dehydro-2-deoxyecdysone | 1.59 | -0.54 | 0.003 | 0.022 | Organic oxides | / |
| Cholesterol sulfate | 1.42 | 0.45 | 0.008 | 0.049 | Steroids and steroid derivatives | Lipid metabolism |

FDR, false discovery rate. KEGG, Kyoto Encyclopedia of Genes and Genomes. VIP, variable importance in projection.
